# Author Correction: Predicting mammalian species at risk of being infected by SARS-CoV-2 from an ACE2 perspective

**DOI:** 10.1038/s41598-021-01576-w

**Published:** 2021-11-04

**Authors:** Yulong Wei, Parisa Aris, Heba Farookhi, Xuhua Xia

**Affiliations:** 1 Department of Biology, University of Ottawa, 30 Marie Curie, Station A, P.O. Box 450, Ottawa, ON K1N 6N5 Canada; 2 Ottawa Institute of Systems Biology, Ottawa, ON K1H 8M5 Canada

Correction to: *Scientific Reports* 10.1038/s41598-020-80573-x, published online 18 January 2021

The original version of this Article contained an error in the Results section, under the subheading ‘Key binding sites on the human ACE2 receptor are most conserved by primates species and variably conserved in selected species belonging to eight other mammalian orders’, where the mink species “*Neovison vison*” was incorrectly given as “*Mustela lutreola*” due to an error in the GenBank SARS-CoV-2 genome records of the host species. It was brought to the attention of the Authors after the publication of this Article that GenBank records of mink-derived SARS-Cov-2 genomes consulted for the original Article (e.g., MT396266.1, MT457398.1, MT457399.1) were not correct at the time of its publication and remain incorrect at the time of publication of this correction notice.

As a result,

“One additional mammal that is known to be infected by SARS-CoV-2 is the mink (*Mustela lutreola*)^18^, a relative of the ferret.”

now reads:

“One additional mammal that is known to be infected by SARS-CoV-2 is the mink (*Neovison vison*)^18^, a relative of the ferret.”

The original Article has been corrected.

